# Genetic Diversity of *Tulipa alberti* and *T. greigii* Populations from Kazakhstan Based on Application of Expressed Sequence Tag Simple Sequence Repeat Markers

**DOI:** 10.3390/plants13182667

**Published:** 2024-09-23

**Authors:** Moldir Yermagambetova, Shyryn Almerekova, Anna Ivashchenko, Yerlan Turuspekov, Saule Abugalieva

**Affiliations:** 1 Institute of Plant Biology and Biotechnology, Almaty 050040, Kazakhstan; ermaganbetova.moldir@bk.ru (M.Y.); almerekovakz@gmail.com (S.A.); yerlant@yahoo.com (Y.T.); 2 Faculty of Biology and Biotechnology, Al-Farabi Kazakh National University, Almaty 050038, Kazakhstan; 3 Institute of Zoology, Almaty 050060, Kazakhstan

**Keywords:** *Tulipa alberti*, *Tulipa greigii*, expressed sequence tag simple sequence repeats, Kazakhstan, genetic diversity, population structure

## Abstract

The genus *Tulipa* L., renowned for its ornamental and ecological significance, encompasses a diversity of species primarily concentrated in the Tian Shan and Pamir-Alay Mountain ranges. With its varied landscapes, Kazakhstan harbors 42 *Tulipa* species, including the endangered *Tulipa alberti* Regel and *Tulipa greigii* Regel, which are critical for biodiversity yet face significant threats from human activities. This study aimed to assess these two species’ genetic diversity and population structure using 15 expressed sequence tag simple sequence repeat (EST-SSR) markers. Leaf samples from 423 individuals across 23 natural populations, including 11 populations of *T. alberti* and 12 populations of *T. greigii*, were collected and genetically characterized using EST-SSR markers. The results revealed relatively high levels of genetic variation in *T. greigii* compared to *T. alberti*. The average number of alleles per locus was 1.9 for *T. alberti* and 2.8 for *T. greigii*. AMOVA indicated substantial genetic variation within populations (75% for *T. alberti* and 77% for *T. greigii*). The Bayesian analysis of the population structure of the two species indicated an optimal value of K = 3 for both species, splitting all sampled populations into three distinct genetic clusters. Populations with the highest level of genetic diversity were identified in both species. The results underscore the importance of conserving the genetic diversity of *Tulipa* populations, which can help develop strategies for their preservation in stressed ecological conditions.

## 1. Introduction

The genus *Tulipa* L., belonging to the family Liliaceae Juss., is globally recognized for its ornamental and economic significance [1]. Taxonomists estimate that the genus comprises between 40 and 140 species worldwide [2,3,4,5,6,7,8,9,10]. The primary centers of gene diversity for tulips are the Tian Shan and Pamir-Alay Mountain ranges in Central Asia [9,11]. Taxonomically, the genus *Tulipa* is divided into four subgenera and 12 sections: *Clusianae* (Baker) Zonn. & Veldkamp (*Clusianae* section), *Orithyia* (D. Don) Baker (*Orithyia*), *Tulipa* L. (*Kolpakowskianae*, *Multiflorae*, *Lanatae*, *Vinistriatae*, *Spiranthera*, *Tulipanum*, and *Tulipa*), and *Eriostemones* (Boiss.) Raamsd (*Sylvestres*, *Biflores*, and *Saxatiles*) [9]. Kazakhstan, characterized by its diverse landscapes and climates, provides a unique habitat for many endemic plant species and is home to 42 species of tulips, which belong to three subgenera: *Orithyia* (1 section, 3 species), *Tulipa* (5 sections, 23 species), and *Eriostemones* (3 sections, 16 species) [12].

Recently, plastid genome sequences have been extensively utilized for species identification and to elucidate molecular plant taxonomy [13,14]. The number of studies on the phylogenetic analysis of *Tulipa* species using nucleotide sequences of the complete plastid genome has increased considerably [15,16]. However, despite these efforts, the taxonomy of the genus remains largely unresolved [10].

Two important species from the *Vinistriatae* section of the *Tulipa* subgenus, *Tulipa greigii* Regel and *Tulipa alberti* Regel, are listed in the Red Book of Kazakhstan [17]. These species thrive in the mountainous regions of Kazakhstan, where they have adapted to specific ecological conditions. However, mass collections for bouquets, excessive grazing, plowing of land, and digging of bulbs pose significant threats to their natural populations in Kazakhstan [17]. Understanding the genetic diversity of these tulip populations is crucial for developing effective conservation strategies.

*T. alberti* is endemic to Kazakhstan, where it inhabits the gravelly and stony slopes found in the foothills and low dry mountains of Sirdarya Karatau, Chu-Ili mountains, Betpakdala, the southwestern spurs of the Zhetysu Alatau, and the western part of the Ili Alatau mountains with sparse vegetation [12]. According to the Red Book of Kazakhstan, this species is considered rare and is classified under category II [17]. *T. greigii* is native to Kazakhstan, Kyrgyzstan, Tajikistan, Uzbekistan, and Iran, usually as single specimens and occasionally in small groups [18,19]. In Kazakhstan, the species is found on the clay and gravelly slopes of the lower mountain ranges in the western and parts of the northern Tian Shan mountains [12]. *T. greigii* is classified as a species with declining numbers, listed under category III in the Red Book of Kazakhstan [17]. E. Regel first described the species *T. alberti* in 1876 and *T. greigii* in 1873, based on specimens collected from the Karatau Mountains [12]. *T. alberti* has high ornamental value and shows great potential for landscape planting and rock gardens [12,17]. *T. greigii* is highly valued and widely used in breeding [12]. Its petals and seed pods are used in traditional medicine, and so are its edible bulbs [12]. Despite the importance of these species, their genetic diversity has not yet been assessed.

Genetic diversity is a crucial aspect of biodiversity and is the foundation for ecosystem and species diversity [20]. Investigating genetic diversity in plant populations is vital for various scientific, ecological, and practical purposes [21]. Furthermore, understanding the genetic diversity of endangered species populations can offer valuable insights for enhancing conservation efforts and optimizing the use of plant resources [22]. Molecular markers can offer valuable insights, especially in research focused on the genetic diversity and population structure of rare and endangered plant species [23]. Among molecular markers, expressed sequence tag simple sequence repeats (EST-SSRs) are widely used markers in population genetic studies [24,25,26]. EST-SSRs are highly transferable across different plant species [27]. They are extensively employed in genetic mapping [28,29] and in evaluating genetic diversity in populations [30,31,32,33,34]. Previous molecular characterizations of the genetic diversity of *Tulipa* populations have employed various markers, including random amplified polymorphic DNA (RAPD) for 10 species of *Tulipa* [35], inter-simple sequence repeat (ISSR) for 39 *Tulipa* accessions [36], single nucleotide polymorphisms (SNPs) for 72 tulip accessions [37], and EST-SSRs for 36 wild and cultivated tulip accessions [38]. Despite the importance of the studied *Tulipa* species and advancements in molecular genetics technology, information about their genetic variation and population genetic structure remains limited.

This study used 15 EST-SSR markers [38,39] to assess the genetic diversity of *T. alberti* and *T. greigii* populations in Kazakhstan. By analyzing genetic markers across multiple populations, we elucidated the levels of genetic variation and identified populations with relatively high diversity. This research enhances our scientific understanding and supports the future preservation activities for these species in their natural habitats.

## 2. Results

### 2.1. Polymorphism of Tested EST-SSR Markers

A total of 11 populations of *T. alberti* and 12 populations of *T. greigii* were genotyped using 15 EST-SSR markers. The genotyping revealed that 9 of the 15 EST-SSRs were polymorphic in the *T. alberti* populations, while 13 showed polymorphism in the *T. greigii* populations. Fifty-two alleles were identified by analyzing 423 samples from 23 populations using 13 EST-SSR markers (Table S1). The number of alleles (Na) per marker varied between three and six, averaging four alleles per locus. The Ca-8508 marker produced the highest number of alleles (six), whereas the markers Ca-6950, Ca-7862, Ca-13333, Kn-2291, and Kn-30956 each generated the lowest number of alleles, with three alleles each. The fragment of the digital electrophoresis for the most polymorphic marker, Ca-8508, is presented in Figure S1. The effective number of alleles ranged between 1.3 (Ca-7862) and 3 (Ca-8508), averaging 1.9. The average genetic diversity (Nei) measured was 0.387. The Shannon diversity index (I) for the studied loci ranged from 0.203 (Ca-7862) to 1.182 (Ca-8508), with an average of 0.608. The average polymorphism information content (PIC) value was 0.560, ranging from 0.195 (Ca-7862) to 0.748 (Ca-8508) (Table 1).

### 2.2. Genetic Diversity in the Collected Populations of Tulipa alberti and Tulipa greigii

The average number of alleles (Na) identified in the 13 EST-SSR polymorphic loci in the 23 study populations was 2.4. The range was from 1.6 (populations 4 and 8 of *T. alberti*) to 3.4 (population 4 of *T. greigii*). The average number of alleles was 1.9 for *T. alberti* populations and 2.8 for *T. greigii* populations. The effective alleles (Ne) ranged from 1.4 for population 4 of *T. alberti* to 2.7 for population 1 of *T. greigii*, averaging 1.9. The polymorphic loci percentage (PPL) averaged 74.6%, varying from 59.4% in *T. alberti* populations to 88.5% in *T. greigii* populations. Nei’s genetic diversity index (h) averaged 0.387, with values ranging from 0.205 in population 8 to 0.353 in population 7 for *T. alberti* and from 0.318 in population 8 to 0.609 in population 1 for *T. greigii*. The populations of *T. greigii* exhibited the highest average h value of 0.487, while *T. alberti* populations had an average index of 0.277. The highest average values for the Ne (2.2) and PPL (88.5%) indices were observed in *T. greigii* populations (Table 2).

### 2.3. Genetic Differentiation and Gene Flow among Populations of Tulipa alberti and Tulipa greigii Species

The analysis of molecular variance (AMOVA) results for the *T. alberti* populations indicated 25% differentiation among populations, aligning with the PhiPT value of 0.248, and 75% of the genetic variation was found within a population. The gene flow (N_m_) was calculated at 0.746 migrants per generation. Analysis was carried out using Fisher’s exact test, treating *p*-values ≤ 0.05 as statistically significant and producing a significant *p*-value (*p* < 0.001). The genetic diversity in *T. greigii* was partitioned as 77% within and 23% among populations. The N_m_ was 1.283 migrants per generation. The PhiPT differentiation index for *T. greigii* was measured at 0.234, demonstrating significant differentiation among populations (Table 3).

The AMOVA results for the total genetic diversity of the two species suggested that 26% of the total genetic variation was among populations and 74% within a population. The PhiPT differentiation index was 0.259, correlating significantly with the percentage of variation observed among the populations. The total gene flow across the two species populations amounted to 1.428 migrants per generation (Table 3).

### 2.4. Genetic Structure of Tulipa alberti and Tulipa greigii Populations

**Figure 1:**
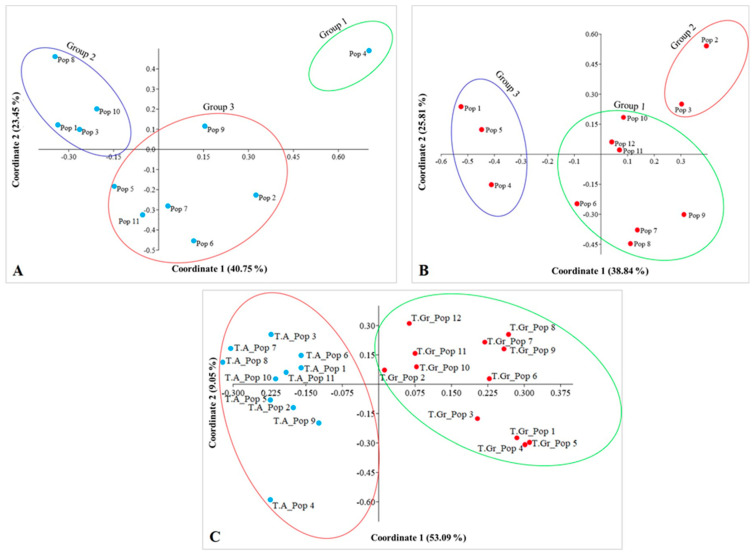
Principal coordinate analysis (PCoA) plots for *Tulipa alberti* populations (**A**); *Tulipa greigii* populations (**B**); and for *Tulia alberti* and *Tulipa greigii* populations (**C**). T.A.—*Tulipa alberti*; T.Gr.—*Tulipa greigii*.

Concerning *T. alberti*, principal coordinate analysis (PCoA) showed that 40.75% and 23.45% of the total genetic variation was explained by the first two coordinates (Figure 1A). For *T. greigii* populations, Coordinate 1 and Coordinate 2 explained 38.84% and 25.81% of the total genetic variation, respectively (Figure 1B). Additionally, the PCoA plot displayed the genetic distances between *T. alberti* and *T. greigii* populations using two principal coordinates (Figure 1C). Coordinate 1 accounted for 53.09% of the total variation among these species’ populations, while Coordinate 2 explained an additional 9.05%. The PCoA plot aids in comprehending the principal patterns of genetic differentiation among the populations under examination (see Figure 1C). Specifically, Coordinate 1 of the PCoA plot effectively differentiates populations of *T. greigii* from those of *T. alberti*, highlighting significant genetic diversity (Figure 1C). This separation indicates that the primary axis of variation captured by Coordinate 1 is crucial for understanding the genetic relationships and divergences between these groups.

**Figure 2:**
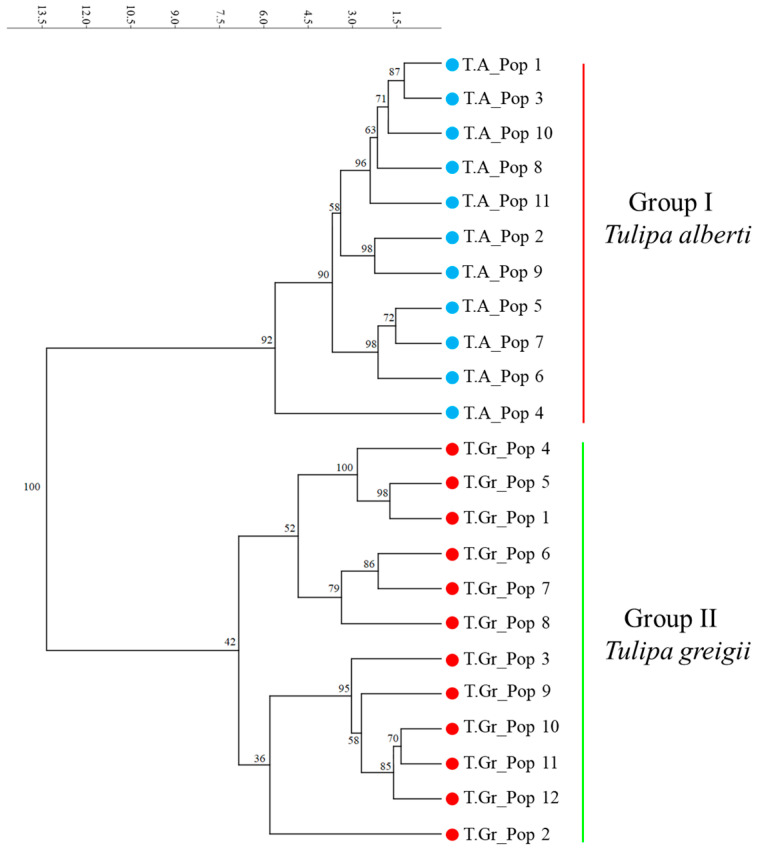
Unweighted pair group method with arithmetic mean (UPGMA) tree constructed from polymorphic EST-SSR loci of *Tulipa alberti and Tulipa greigii* populations. T.A.—*Tulipa alberti*; T.Gr.—*Tulipa greigii*.

Genetic diversity in 11 *T. alberti* and 12 *T. greigii* populations was assessed using an unweighted pair group method with arithmetic mean (UPGMA) method. The resulting dendrogram separated the studied populations into two distinct groups: Group I, consisting exclusively of *T. alberti* populations, and Group II, containing populations of *T. greigii* (Figure 2). The results of the UPGMA clustering analysis are consistent with the differentiation observed in the PCoA plot (Figure 1).

The Mantel correlation coefficient r^2^ = 0.0147 (*p* < 0.001) indicated a statistically significant but weak correlation between genetic distance and geographic distance matrices among populations in the entire *T. alberti* and *T. greigii* datasets. Additionally, the contribution of altitude to genetic differentiation was found to be statistically non-significant.

**Figure 3:**
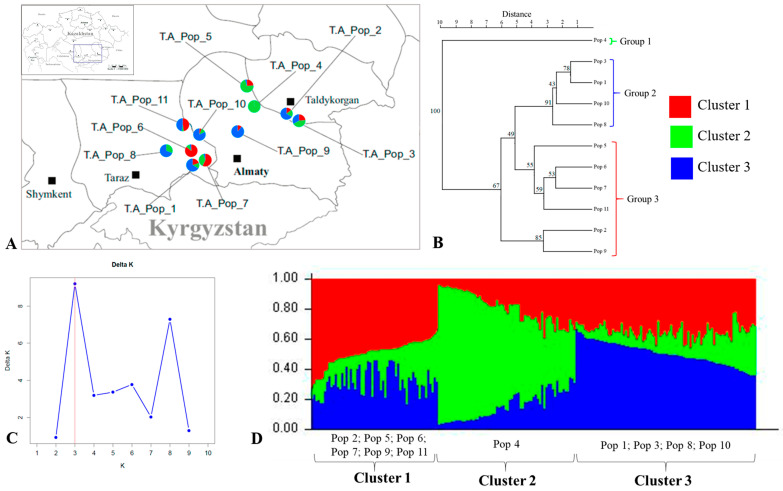
Genetic structure of *Tulipa alberti* (T.A.) populations. Distribution of *Tulipa alberti* populations (**A**); UPGMA tree of *Tulipa alberti* populations (**B**); STRUCTURE analysis graphic with the Evanno method showing optimal K = 3 (**C**); and Bayesian inference clustering of 207 individuals from 11 *Tulipa alberti* populations (**D**).

Genetic structure analyses grouped all *T. alberti* populations into three clusters. Bayesian structure analysis confirmed an optimal K value of 3, classifying 207 individuals from 11 populations into three main genetic clusters (Figure 3). Additionally, UPGMA results revealed three groups of *T. alberti* populations, corresponding to the three clusters identified by the STRUCTURE analysis (Figure 3B). Specifically, Group 1 included population 4, which corresponds to Cluster 2; Group 2 consisted of the larger number of individuals from populations 1, 3, 8, and 10 (Cluster 3); and Group 3 encompassed individuals from the remaining populations 2, 5, 6, 7, 9, and 11. The results indicated a moderate level of genetic structure among the *T. alberti* populations (Figure 3).

**Figure 4:**
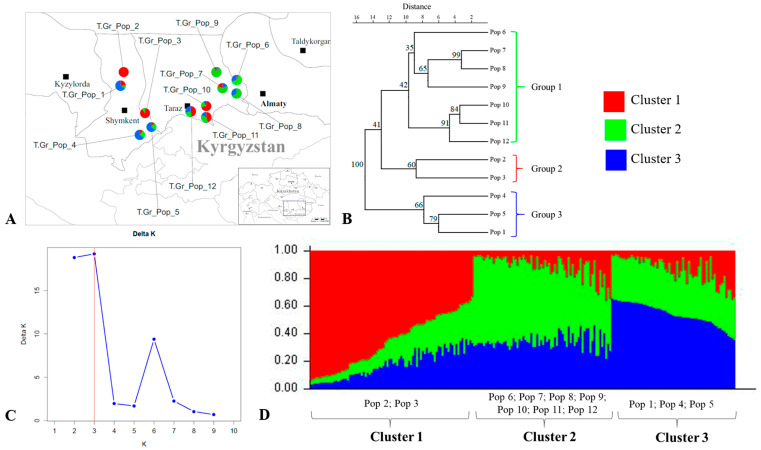
Genetic structure of *Tulipa greigii* (T.Gr.) populations. Distribution of *T. greigii* populations (**A**); UPGMA tree of *T. greigii* populations (**B**); STRUCTURE analysis graphic with the Evanno method showing optimal K = 3 (**C**); and Bayesian inference clustering of 216 individuals from 12 *T. greigii* populations (**D**).

The Bayesian structure analysis of *T. greigii* populations also indicated an optimal value of K = 3 for clustering the *T. greigii* population into three groups (Figure 4). For the *T. greigii* populations, the UPGMA clustering results were consistent with the findings from the STRUCTURE analysis (Figure 4B). Group 1 includes populations 6, 7, 8, 9, 10, 11, and 12 from the southeast region of Kazakhstan (Figure 4A), corresponding to Cluster 2. Populations from the Turkestan region (Figure 4A) were divided into two groups: Group 2, comprising populations 2 and 3, corresponds to Cluster 1, while Group 3, consisting of populations 1, 4, and 5, aligns with Cluster 3.

The results from STRUCTURE and UPGMA analyses of *T. alberti* and *T. greigii* populations agreed with the findings from the PCoA analysis (Figure 1).

## 3. Discussion

The present study used polymorphic EST-SSR markers to elucidate the genetic diversity and population structure of Kazakhstan’s *T. alberti* and *T. greigii species*. The results provide significant perceptions of the genetic variation within and between populations of these species, contributing valuable information for their conservation and management. The polymorphism detected in 9 out of 15 EST-SSR markers for *T. alberti* and 13 EST-SSR markers for *T. greigii* highlights a relatively higher genetic variability in *T. greigii* populations. The average number of alleles per locus and the effective number of alleles were found to be higher in *T. greigii* compared to *T. alberti*. This suggests a greater genetic diversity within *T. greigii* populations. Given that *T. alberti* is a locally endemic species, these results align with the findings of Ferrer et al. [40] and Shen et al. [41], who reported higher genetic variation in species with broader geographical distributions and more diverse habitats. The informativeness of a marker can be quantitatively assessed using the PIC value, which is influenced by the number of detectable alleles and their frequency distribution [42]. The DNA marker is considered informative when the PIC value is ≥0.5 [43]. In this study, the mean PIC value of 0.56 (Table 1) indicates that the EST-SSR markers were nearly highly informative compared with ISSR [44], RAPD [45], and AFLP [46] markers and had sufficient discriminatory power to assess genetic diversity. The highest PIC value was observed in the Ca-8508 marker and the lowest in the Ca-5553 marker for *T. alberti* populations (Table 1). In contrast, for *T. greigii* populations, the Ca-8508 marker also exhibited the highest PIC value, while the Ca-7862 marker showed the lowest (Table 1). The average PIC for both species’ populations revealed that the markers Ca-8508, Ca-15730, and Ca-5526 exhibited the highest values. This finding aligns with previous studies, particularly noting that the Ca-15730 marker consistently demonstrated the highest PIC value in wild and cultivated tulip populations [38,47].

The AMOVA results demonstrated that a substantial proportion of the genetic variation in *T. alberti* (75%) and *T. greigii* (77%) is found within populations, with less variation observed between populations (Table 3). This intrapopulation genetic variation indicates significant gene flow and genetic exchange within populations. However, the lower gene flow in *T. alberti* (0.746 migrants per generation) compared to *T. greigii* (1.83 migrants per generation) suggests that *T. alberti* populations are more genetically isolated, which could be due to its more restricted habitat range and smaller population sizes. The PCoA plot (Figure 1) illustrated significant genetic differentiation between *T. greigii* and *T. alberti* populations. Coordinate 1, explaining 53.09% of the variation, effectively separates the species. This finding is supported by an SSR-based UPGMA dendrogram (Figure 2), which also clusters populations into distinct *T. greigii* and *T. alberti* groups. The congruence between PCoA and UPGMA analyses underscores robust genetic distinctions between these *Tulipa* species, providing insights into their evolutionary divergence and genetic structure. The distinct clustering of *T. greigii* and *T. alberti* populations in the PCoA plot and the separation observed in the UPGMA dendrogram were consistent with the significant genetic differentiation indicated by the PhiPT values. These results corroborate previous phylogenetic studies that have identified clear genetic boundaries between plant species [48,49,50]. The Mantel test revealed a weak but significant correlation between genetic and geographic distances, suggesting that geographic isolation has some influence on genetic differentiation. At the same time, the contribution of altitude to genetic differentiation was found to be non-significant, indicating that other ecological and evolutionary factors play a more crucial role in shaping the genetic structure of these populations.

The genetic structure analysis of both *T. alberti* and *T. greigii* populations revealed a moderate level of genetic differentiation among the sampled populations, with an optimal K value of 3, indicating the presence of three distinct genetic clusters within each species. The consistent results from Bayesian STRUCTURE analysis, UPGMA clustering, and PCoA provide robust evidence for genetic subdivision within these species, corroborating findings from studies on other species [51,52,53]. Various genetic diversity parameters (Ne, I, h, and %P) revealed that a population of *T. greigii* (population 1) collected in the mountainous areas of the Karatau ridge, located in northwestern Tian Shan in Kazakhstan, exhibited the highest levels of genetic diversity (Table 2). Consequently, the Karatau can be accepted as one of the centers of genetic diversity for *T. greigii*. Relatively homogeneous diversity can also be observed from the core to the periphery (from the western to the eastern populations), suggesting a relatively high gene flow (1.283), which supports Mayr’s hypothesis [54]. This finding is consistent with previous studies on different plant species [55,56]. High genetic diversity indices were found in population 7 of *T. alberti* (Table 2), collected in Zhetyzhol ridge (Zhambyl region), the western part of the Trans-Ili Alatau. This population might represent the origin of the species, exhibiting homogeneous genetic diversity across its range with a gene flow value of 0.746. Notably, this species is endemic to Kazakhstan [12], highlighting the importance of the location of population 7 for future conservation studies.

The genetic analysis of *T. alberti* and *T. greigii* populations revealed significant genetic diversity and differentiation between these species. The higher genetic diversity and gene flow in *T. greigii* suggest a more dynamic and interconnected population structure, while the lower genetic diversity and gene flow in *T. alberti* indicate more isolated and genetically distinct populations. Overall, these results identifying the most genetically diverse populations within these two species can be efficiently used to develop conservation strategies and manage these species in natural habitats.

## 4. Materials and Methods

### 4.1. Plant Material and DNA Extraction

**Figure 5:**
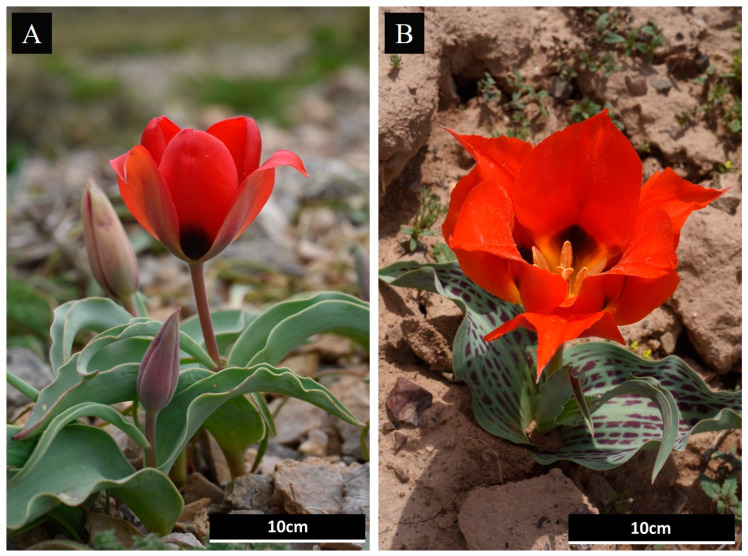
*Tulipa alberti* (**A**) and *Tulipa greigii* (**B**) species in nature. Photos were taken by Oleg Belyalov.

During their blooming period, from early April to late May, a total of 423 individuals from 23 natural populations of two *Tulipa* species were sampled: 11 populations of *T. alberti* (Figure 5A) and 12 populations of *T. greigii* (Figure 5B).

**Figure 6:**
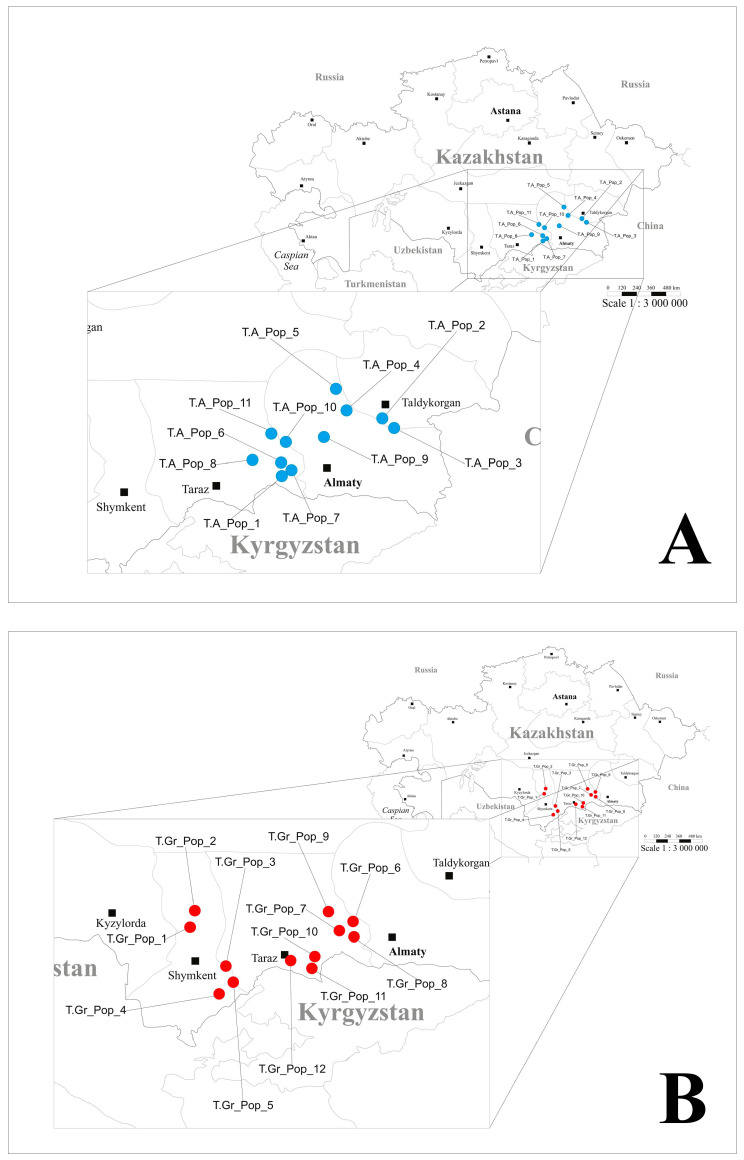
Location of sampled *Tulipa alberti* (**A**) and *Tulipa greigii* (**B**) populations in Kazakhstan. Pop—population; numeration according to Table 1. T.A.—*Tulipa alberti*; T.Gr.—*Tulipa greigii*.

These populations were located in the southern and southeastern regions of Kazakhstan (Figure 6), at altitudes ranging from 620 m (*T. alberti*, population 9) to 1817 m (*T. greigii*, population 4) above sea level. In each population, leaves were collected from 6 to 21 randomly selected mature plants spaced at least 10 m apart to minimize sampling of genetically identical individuals. The number of collected samples in each population varied according to the sizes of natural populations (Table 4).

The geographical coordinates and altitudes of the populations were documented using a handheld Garmin Etrex GPS device (Taiwan, China). Permission to collect plant leaves from the Red Book species *T. alberti* and *T. greigii* was provided by the Forestry and Wildlife Committee of the Ministry of Ecology, Geology, and Natural Resources of the Republic of Kazakhstan. Details of the populations, including collection sites and altitude, are provided in Table 4. Leaf samples for DNA analysis were immediately placed in labeled, sealed bags with silica gel to ensure desiccation.

Total genomic DNA was extracted from dried young leaves using the cetyltrimethylammonium bromide (CTAB) method described by Doyle [57]. The quality and quantity of the DNA were assessed through spectrophotometry and by visually comparing DNA dilutions run on a 1% agarose gel. The DNA sample concentrations were normalized to 100 ng/μL and stored at −20 °C for subsequent EST-SSR analysis.

### 4.2. EST-SSR Analysis

In this study, a total of 15 EST-SSR markers [38,39] were utilized. Details about the primer sequences, repeat motifs, and amplification conditions for the tulip microsatellites are given in Table 5.

The PCR amplification conditions were set as follows: An initial denaturation occurred at 94 °C for 5 min, followed by 30 cycles of 94 °C for 30 s, 57 °C for 45 s, and 72 °C for 35 s. This was followed by eight cycles of 94 °C for 30 s, 54 °C for 45 s, and 72 °C for 35 s, with a final extension at 72 °C for 8 min. The PCR reactions were carried out in a 20 μL mixture comprising 1× PCR buffer, 1.5 mM MgCl_2_, 0.5 mM of each dNTP, 0.2 μM of each primer, 1 unit of Taq DNA polymerase, and 100 ng of DNA template. Separation of PCR products was achieved using the QIAxcel Connect System capillary electrophoresis (QIAGEN, Hilden, Germany) equipped with the QIAxcel DNA High Resolution Kit (QIAGEN, Hilden, Germany) and QX Alignment Marker (QIAGEN, Hilden, Germany) (15 bp/3 kb).

### 4.3. Statistical Analysis

The genetic diversity and structure parameters, including the number of alleles (Na), the effective number of alleles (Ne), Shannon’s information index (I), percentage of polymorphic loci (PPL), Nei’s genetic diversity index (Nei), principal coordinate analysis (PCoA), and the analysis of molecular variance (AMOVA) were assessed using the GenAlEx 6.5 software [58]. Additionally, the amount of gene flow (N_m_) between gene pools was determined based on F_ST_ estimates using the formula Nm = [(1/F_ST_) − 1]/4. The polymorphism information content (PIC) was computed using iMEC software (https://irscope.shinyapps.io/iMEC/, accessed on 22 May 2024) [59].

The dendrogram of a total of 23 populations of *T. alberti* (11 populations) and *T. greigii* (12 populations) was reconstructed using the unweighted pair group method with arithmetic means (UPGMA) based on genetic distance matrices, employing the PAST 4.03 software [60] with 1000 bootstrap replications. The correlations between genetic and geographic distance (Mantel test) were estimated for all populations by GenAlEx 6.5 software [58]. For analyzing the genetic structure of the populations of *Tulipa greigii* and *Tulipa alberti*, the STRUCTURE v2.3.4 software was utilized, applying the Bayesian clustering method [61]. The settings for the burn-in period and the Markov chain Monte Carlo (MCMC) replications were established at 100,000, with the number of iterations set to 3. The results from the STRUCTURE analysis were assessed using the web-based program Structure Harvester, which utilizes the methods of Evanno et al. [62] and Jakobsson and Rosenberg [63] to determine the optimal number of genetic clusters (K).

## 5. Conclusions

Using the application of 15 EST-SSR markers, this study provided valuable insights into the genetic diversity and population structure of Red Book species *T. greigii* and *T. alberti* in Kazakhstan. The relatively high genetic diversity and gene flow in *T. greigii* populations suggested a more dynamic population structure. In contrast, low genetic diversity and gene flow in *T. alberti* indicated more isolated and genetically distinct populations. The populations of *T. greigii* in the Karatau ridge and *T. alberti* in the Zhambyl region exhibited relatively high genetic diversity compared to other studied populations within these two species. The results of this study can provide valuable insights for the development of conservation strategies aimed at preserving the genetic diversity of these endangered species.

## Figures and Tables

**Table 1:** Characteristics of 13 polymorphic microsatellite markers used in this study.

Species	EST-SSRs	N	Na	Ne	I	h	PIC
*Tulipa alberti*	Ca-2572	207	3	2.4	0.959	0.581	0.578
	Ca-3952	207	1	1.0	0.000	0.000	0.000
	Ca-5526	207	2	1.8	0.643	0.453	0.451
	Ca-5553	207	4	1.7	0.692	0.418	0.416
	Ca-6950	207	2	1.9	0.673	0.483	0.480
	Ca-7862	207	1	1.0	0.000	0.000	0.000
	Ca-8508	207	4	3.4	1.286	0.707	0.703
	Ca-13333	207	3	2.3	0.946	0.565	0.562
	Ca-15730	207	3	1.9	0.760	0.473	0.471
	Kn-834	207	4	2.9	1.118	0.661	0.658
	Kn-2291	207	2	1.8	0.637	0.447	0.444
	Kn-7480	207	1	1.0	0.000	0.000	0.000
	Kn-30956	207	1	1.0	0.000	0.000	0.000
	Mean	207	2.4	1.9	0.593	0.368	0.366
	SE		0.331	0.209	0.126	0.075	0.267
*Tulipa greigii*	Ca-2572	216	5	2.3	1.099	0.572	0.569
	Ca-3952	216	4	2.5	1.118	0.599	0.597
	Ca-5526	216	5	3.8	1.371	0.737	0.734
	Ca-5553	216	4	2.0	0.917	0.501	0.499
	Ca-6950	216	3	1.8	0.747	0.432	0.430
	Ca-7862	216	3	1.7	0.703	0.397	0.395
	Ca-8508	216	6	4.5	1.603	0.782	0.779
	Ca-13333	216	2	2.0	0.690	0.499	0.497
	Ca-15730	216	5	4.0	1.471	0.753	0.750
	Kn-834	216	3	2.0	0.872	0.506	0.504
	Kn-2291	216	3	2.5	0.997	0.610	0.607
	Kn-7480	216	4	3.3	1.289	0.704	0.700
	Kn-30956	216	3	2.3	0.920	0.567	0.564
	Mean	216	3.8	2.7	1.061	0.589	0.586
	SE		0.317	0.257	0.082	0.034	0.124
Total	Ca-2572	423	5	2.0	0.685	0.431	0.567
	Ca-3952	423	4	1.5	0.368	0.240	0.616
	Ca-5526	423	5	1.9	0.648	0.424	0.706
	Ca-5553	423	4	1.6	0.513	0.309	0.676
	Ca-6950	423	3	1.7	0.578	0.398	0.467
	Ca-7862	423	3	1.3	0.203	0.135	0.195
	Ca-8508	423	6	3.0	1.182	0.688	0.748
	Ca-13333	423	3	1.7	0.555	0.379	0.584
	Ca-15730	423	5	2.5	0.941	0.572	0.714
	Kn-834	423	4	2.1	0.751	0.496	0.598
	Kn-2291	423	3	1.8	0.647	0.439	0.558
	Kn-7480	423	4	1.8	0.465	0.275	0.453
	Kn-30956	423	3	1.5	0.369	0.245	0.398
	Mean	423	4	1.9	0.608	0.387	0.560
	SE		0.065	0.047	0.026	0.016	0.152

Notes: N—number of samples; Na—number of alleles per locus; Ne—effective number of alleles; I—Shannon’s information index; h—Nei’s genetic diversity index; PIC—polymorphism information content; SE—standard error.

**Table 2:** Assessment of the genetic diversity of studied *Tulipa* species populations.

Species	Population ID	Na	Ne	I	h	PPL
*Tulipa alberti*	T.A. Pop 1	1.8	1.5	0.414	0.277	69.23%
	T.A. Pop 2	2.0	1.7	0.465	0.299	61.54%
	T.A. Pop 3	1.8	1.6	0.439	0.300	61.54%
	T.A. Pop 4	1.6	1.4	0.315	0.219	46.15%
	T.A. Pop 5	1.9	1.5	0.413	0.274	69.23%
	T.A. Pop 6	2.1	1.7	0.488	0.312	61.54%
	T.A. Pop 7	2.0	1.8	0.529	0.353	69.23%
	T.A. Pop 8	1.6	1.4	0.303	0.205	46.15%
	T.A. Pop 9	2.0	1.6	0.441	0.274	61.54%
	T.A. Pop 10	1.8	1.5	0.380	0.258	53.85%
	T.A. Pop 11	1.8	1.6	0.429	0.280	53.85%
	Mean	1.9	1.6	0.420	0.277	59.4%
	SE	0.154	0.121	0.068	0.041	0.085
*Tulipa greigii*	T.Gr. Pop 1	3.2	2.7	0.993	0.609	100.00%
	T.Gr. Pop 2	2.6	1.9	0.638	0.392	69.23%
	T.Gr. Pop 3	2.7	2.2	0.731	0.439	69.23%
	T.Gr. Pop 4	3.4	2.4	0.947	0.558	100.00%
	T.Gr. Pop 5	3.3	2.4	0.929	0.558	100.00%
	T.Gr. Pop 6	2.9	2.3	0.822	0.495	84.62%
	T.Gr. Pop 7	2.6	2.0	0.747	0.481	100.00%
	T.Gr. Pop 8	1.8	1.5	0.406	0.318	69.23%
	T.Gr. Pop 9	2.3	1.8	0.615	0.410	84.62%
	T.Gr. Pop 10	2.9	2.2	0.830	0.513	92.31%
	T.Gr. Pop 11	2.8	2.2	0.804	0.529	100.00%
	T.Gr. Pop 12	3.2	2.4	0.904	0.548	92.31%
	Mean	2.8	2.2	0.781	0.487	88.5%
	SE	0.466	0.323	0.167	0.084	0.129
Total	Mean	2.4	1.9	0.608	0.387	74.6%
	SE	0.065	0.047	0.026	0.016	3.82%

Notes: T.A.—*Tulipa alberti*; T.Gr.—*Tulipa greigii*; Pop—population; Na—number of alleles per locus; Ne—effective number of alleles; I—Shannon’s information index; h—Nei’s genetic diversity index; PPL—the percentage of polymorphic loci; SE—standard error.

**Table 3:** The analysis of molecular variance (AMOVA) based on Nei’s genetic distance in populations of *Tulipa alberti* and *Tulipa greigii*.

Species	Source	df	SS	MS	Est.Var.	%	PhiPT	Nm	*p* Values
*Tulioa alberti*	Among Pops	10	197.049	19.705	0.904	25%			
	Within Pops	196	536.796	2.739	2.739	75%			
	Total	206	733.845		3.642	100%	0.248	0.746	<0.001
*Tulipa greigii*	Among Pops	11	512.880	46.625	2.204	23%			
	Within Pops	204	1468.755	7.200	7.200	77%			
	Total	215	1981.634		9.403	100%	0.234	1.283	<0.001
Total	Among Pops	1	234.507	234.507	1.094	26%			
	Within Pops	421	1316.394	3.127	3.127	74%			
	Total	422	1550.901		4.221	100%	0.259	1.428	<0.001

Notes: df—degrees of freedom; SS—sum of squares; MS—mean squared; Est.Var.—estimates of variance; %—percentage of variation; PhiPT—genetic differentiation index among population; Nm—gene flow (Nm) value.

**Table 4:** The locations of populations of two tulip species collected in the southern and southeastern regions of Kazakhstan.

Species	Population ID	Sample Size	Altitude	Collection Sites
*Tulipa alberti*	T.A. Pop 1	15	1100	Zhetyzhol ridge, western part of the Trans-Ili Alatau, Zhambyl region
	T.A. Pop 2	21	600	Qonayev district, Trans-Ili Alatau, Almaty region
	T.A. Pop 3	21	611	Chulak Mountains, Trans-Ili Alatau, Almaty region
	T.A. Pop 4	17	510	The right bank of the Ili River, in the area of the Kapchagai reservoir, gravelly slopes, Trans-Ili Alatau, Almaty region
	T.A. Pop 5	21	575	Malaysary pass, Trans-Ili Alatau, Almaty region
	T.A. Pop 6	21	1064	Zhetyzhol ridge, eastern slope, Kenen village, western part of the Trans-Ili Alatau, Zhambyl region
	T.A. Pop 7	21	1072	Zhetyzhol ridge, southwestern slope, Kenen village, western part of the Trans-Ili Alatau, Zhambyl region
	T.A. Pop 8	13	700	Right bank of the Tarylgan river, Chu-Ili mountains, Zhambyl region
	T.A. Pop 9	19	620	Kurtinsky district, near the Kurtinsky reservoir, Chu-Ili mountains, Almaty region
	T.A. Pop 10	17	870	Tamgaly tas, Chu-Ili mountains, Almaty region
	T.A. Pop 11	21	880	Anrakai Mountains, Chu-Ili mountains, Zhambyl region
*Tulipa greigii*	T.Gr. Pop 1	21	1054	Kazanbulak tract, Karatau ridge, Karatau Nature Reserve, Turkestan region
	T.Gr. Pop 2	20	1033	Arpaozen, Kekliktas tract, Karatau ridge, Karatau Nature Reserve, Turkestan region
	T.Gr. Pop 3	20	932	Shubaykyzyl hilly area, Tyulkubas district, Turkestan region
	T.Gr. Pop 4	21	1817	Kaskasu gorge, Mailoshak ridge, Sairam-Ugam state national nature park (SNNP), Turkestan region
	T.Gr. Pop 5	21	1613	Iirsu village, Aksu Valley, Aksu-Zhabagly state nature reserve SNR, Turkestan region
	T.Gr. Pop 6	20	970	Kordai Pass, eastern slope, Trans-Ili Alatau, Zhambyl region
	T.Gr. Pop 7	21	1270	Kordai Pass, southeast slope, right side of the road, Trans-Ili Alatau, Zhambyl region
	T.Gr. Pop 8	6	1120	Kordai Pass, southeast slope, left side of the road, Trans-Ili Alatau, Zhambyl region
	T.Gr. Pop 9	15	1190	On the right side of the road towards Taraz, western tip of the Trans-Ili Alatau, Zhambyl region
	T.Gr. Pop 10	21	1010	Merken district, Merke gorge, northern slope, Kyrgyz Alatau, Zhambyl region
	T.Gr. Pop 11	11	1260	Merken district, Merke gorge, Kyrgyz Alatau, Zhambyl region
	T.Gr. Pop 12	21	1140	Almalysay gorge, western tip of the Kyrgyz Alatau, Zhambyl region

**Table 5:** Characteristics of EST-SSR markers used for analysis of *Tulipa* species.

№	Locus	Repeat Motif	Primer Sequence (5′–3′)	Product Size (bp)
1	Ca-2572	(GAGAAG)4	F-TGCACAGAGCCAAAGAAGTA R-TCTCCTTTCCATGTTTCCTC	213
2	Ca-3952	(CAG)4	F-ACTCAATTCACTTGCAGCAG R-GTCGTTGCAGTTGTTGTGAT	189
3	Ca-5526	(GAG)6	F-TTTACGGGAATTACTTCGAG R-ACATGGATTCCAAACAAGAG	242
4	Ca-5553	(TTG)9	F-CCGATAATTGAGGTCAGGTT R-CCGAACTCCTCGCATATAAC	168
5	Ca-6950	(GAT)4	F-ATGCAATCTTGGGAACTGAT R-CACTGTCGTCATCTTCTCCA	198
6	Ca-7862	(CGC)4	F-AATCAACGCATCATGTCAAC R-TACTGGAGGTACGCCTCCTT	131
7	Ca-8508	(GTT)10	F-AGAATTTGTCTTGCGACAGT R-TAGGGGTACCAATTTGTGTT	325
8	Ca-13333	(GAT)4	F-ATGGTTGGAAGAGGAGACTG R-AGTCATTCGATCCTCGAGTC	242
9	Ca-15730	(CGC)8	F-CATCAAAACCGACAACACC R-CGGTCAACATCATTCAAGAG	213
10	Kn-834	(AT)8	F-TCAGAAGGCTCTTCTTTCAG R-CTTTACATGGAGATAATGTTAACAA	221
11	Kn-1412	(GGA)10	F-GTCCTTTGTACGGTGATGTT R-TAGCTTCCGGAGTTCAATAG	242
12	Kn-2291	(GAG AAG)4	F-GAAGACGAAGATGATTCGAG R-TGGGTTTCACTTAAACAGCT	275
13	Kn-7108	(TTTC)4	F-TTGCTGCTTCGACTACTTTG R-GGTCATGCAACATAAACTGC	231
14	Kn-7480	(GAC)9	F-GCAACTTAGGTCAACAGAGG R-CTCCTACCAACAAAGCATTC	268
15	Kn-30956	(CTC)6	F-TGAAGCTCCTCCACTCTACC R-ACAAGGGCACTCATTCTGTT	237

## Data Availability

Data are contained within the article and Supplementary Materials.

## Supplementary Materials

The following supporting information can be downloaded at https://www.mdpi.com/article/10.3390/plants13182667/s1: Figure S1: The fragment of the digital electrophoresis for the most polymorphic Ca-8508 marker; Table S1: Allelic status in fifteen SSR markers for *Tulipa greigii* and *Tulipa alberti* populations (bp).
