# Supplementary figures and images for: Genetic Diversity of Tulipa alberti and T. greigii Populations from Kazakhstan Based on Application of Expressed Sequence Tag Simple Sequence Repeat Markers
Source: Plants (Basel). 2024 Sep 23;13(18):2667. doi: 10.3390/plants13182667 (PMC11435150; doi:10.3390/plants13182667)

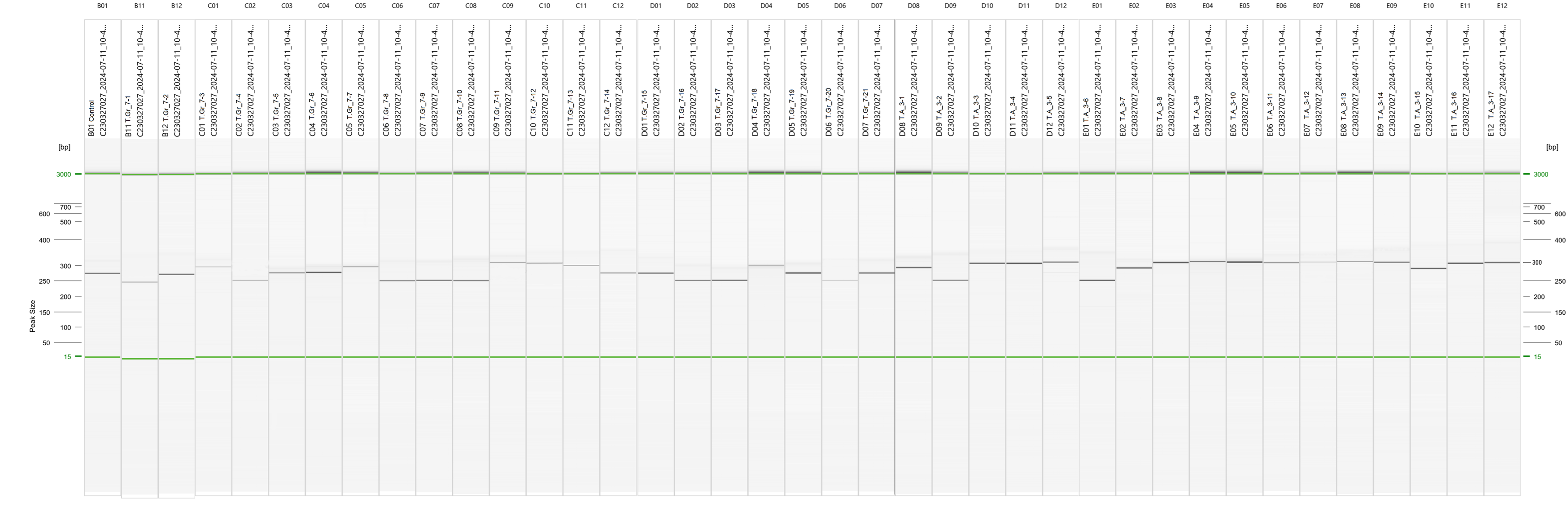

Supplement: Supplementary file 1 [file plants-13-02667-s001.zip › Supplementary Materials/Figure S1.png]
